# Fatal Inferior STEMI Post Left Heart Catheterization

**DOI:** 10.51894/001c.164418

**Published:** 2026-08-01

**Authors:** Abdallah Almawazreh, Suliman Alhassan, Kamal Khalil

**Affiliations:** 1 Detroit Medical Center - Sinai Grace Hospital

## 56

### Background

Left heart catheterization (LHC) is widely performed to evaluate suspected coronary artery disease (CAD) in patients with ischemic symptoms or abnormal findings on electrocardiography, echocardiography, or stress testing. Although complication rates have declined with advances in technique, new ST-segment elevation myocardial infarction (STEMI) after LHC remains a rare but serious event. Proposed mechanisms include coronary dissection, thromboembolism, vasospasm, or catheter-related thrombosis. We describe a fatal case of STEMI shortly after LHC.

### Case Description

A 77-year-old woman with heart failure with reduced ejection fraction, hypertension, type 2 diabetes mellitus, and prior pulmonary embolism presented with crushing substernal chest pain radiating to the left arm, associated with dyspnea and nausea. Electrocardiogram showed T-wave inversions in V1–V3. High-sensitivity troponin was 184 ng/L and BNP 643 pg/mL. She received aspirin and underwent urgent LHC, which revealed luminal irregularities without obstructive CAD. Following sheath removal, she developed lethargy and profound sinus bradycardia, responding to atropine, and was transferred to the ICU. Soon after, she developed recurrent chest pain and hemodynamic instability. Repeat ECG showed new inferior ST-segment elevations consistent with acute inferior STEMI. She developed pulmonary edema requiring noninvasive ventilation. Troponin levels rose markedly, peaking above 22,973 ng/L. Therapeutic heparin was initiated. Transthoracic echocardiography demonstrated a left ventricular ejection fraction of 25–30% with inferior and posterior wall akinesia. A 1.13-cm echogenic mass on the right coronary cusp of the aortic valve raised concern for thrombus. Transesophageal echocardiography was planned but not completed due to clinical deterioration. The patient developed pulseless electrical activity followed by refractory ventricular fibrillation. Despite advanced cardiac life support, resuscitation was unsuccessful.

### Discussion

STEMI after LHC is uncommon but potentially fatal, particularly when initial angiography shows no obstructive disease. Coronary embolization or catheter-induced thrombosis were suspected, with possible valvular thromboembolism suggested by the aortic valve mass. Early bradycardia may have reflected evolving inferior ischemia. This case underscores the need for vigilant post-procedural monitoring and rapid reassessment of new ischemic changes, as outcomes can be catastrophic despite prompt intervention.

